# Perceived changes in extreme weather among older people – insights from Austria

**DOI:** 10.3389/fpsyg.2026.1794515

**Published:** 2026-05-05

**Authors:** Jasmin Riederer, Lukas Richter

**Affiliations:** 1 Karl Landsteiner Privatuniversitat fur Gesundheitswissenschaften GmbH, Krems an der Donau, Austria; 2 Hochschule für Angewandte Wissenschaften St. Pölten GmbH, St. Pölten, Austria; 3 Institute for Sociology and Social Research, Vienna University of Economics and Business, Vienna, Austria

**Keywords:** Austria, climate gerontology, extreme weather, older adults, risk perception, SHARE

## Abstract

**Background:**

Climate gerontology examines the intersections between climate change and population ageing, yet empirical evidence on how older adults perceive climate-related environmental change remains limited, particularly in European contexts. This study analyses perceptions of changes in the intensity and frequency of extreme weather events among people aged 50 and over in Austria and investigates the social factors associated with these perceptions.

**Methods:**

Using data from the Austrian subsample of the 2021/2022 wave of the Survey of Health, Ageing and Retirement in Europe (SHARE; *N* = 3,170), an index of perceived changes in weather extremes since childhood was constructed. That was used to estimate a hierarchical multiple linear regression model including environmental awareness, sociodemographic characteristics, financial strain, subjective health, loneliness, and urbanicity.

**Results:**

The results indicate that perceptions of increasing extreme weather are widespread among older adults in Austria, pointing to a broadly shared awareness of climate-related change. At the same time, perceptions are socially and spatially differentiated. Environmental awareness emerges as the strongest correlate of perceived increases, underscoring the importance of cognitive and informational factors in shaping climate-related interpretations. Urban residence is associated with stronger perceptions compared to town and rural living, suggesting the relevance of geographic context and everyday exposure. Higher education and financial hardship are also positively associated with perceived increases. In contrast, subjective health and loneliness show no significant associations.

**Conclusion:**

Overall, the findings suggest that perceptions of extreme weather among older adults are structured by social position, urbanicity, and environmental awareness. These results call for differentiated climate communication and adaptation strategies.

## Introduction

Over the last decade, global mean temperatures have been approximately 1.25 °C above pre-industrial levels (Forster et al., 2025), leading to extreme heat and rainfall events that can be traced back to anthropogenic climate change (Robinson et al., 2021). In Austria, for example, the year 2024 saw record-breaking summer temperatures, followed by flooding across several regions (Nature Climate Change, 2024). As the effects of climate change become more tangible, it is becoming increasingly evident that different societal groups are affected by extreme weather events in different ways (Wrotek et al., 2025). Vulnerability to such events depends strongly on age, socio-economic factors and community cohesion (Babcicky and Seebauer, 2021). While wealthier households have more adaptive resources and greater recovery capacity, low-income individuals are more exposed and have fewer coping mechanisms (Cutter et al., 2003; Thomas et al., 2019). Social isolation further compounds these risks, whereas strong community networks enhance collective resilience (Thomas, 2016).

Older adults are among the most vulnerable demographic groups to the effects of climate change (Choi et al., 2023; Tipaldo et al., 2024). Physical limitations related to age or chronic health conditions make older individuals particularly vulnerable during extreme weather events (IPCC, 2014; Rhoades et al., 2018). Key climate hazards that disproportionately endanger older adults include heatwaves, floods, wildfires, droughts and storms. These hazards can exacerbate cardiovascular diseases and mental stress while reducing overall quality of life (Zhang et al., 2025). “Climate change not only directly impacts older people’s longevity but also healthy aging, which is the process of maintaining physical and mental capacities while optimizing functional abilities” (Prina et al., 2024, 1). This growing concern has given rise to the emerging interdisciplinary field of climate gerontology (Haq and Gutman, 2014), which examines the intersection of climate change and the specific vulnerabilities (Rhoades et al., 2018), coping strategies (Khalil et al., 2025) and lived experiences of older populations (Budziszewska, 2024). However, older adults are not merely passive victims of climate change. As emphasised by Zhang and Chen (2024), they also possess significant social capital, life experience, and knowledge that can contribute to adaptation and resilience strategies. Furthermore, the growing older adult population contributes significantly to climate change through its consumption and behavior (Zheng et al., 2022).

In other words, the belief in climate change among older people is becoming an important factor in the fight against it. Therefore, it is essential to understand the factors contributing to this belief. One such factor is the perception of extreme weather, which influences preparedness, risk perception, adaptive behavior and also general beliefs about climate change (Lujala and Lein, 2020; Spence et al., 2012). Although the strength of the correlation varies (Hughes et al., 2020; Kleespies et al., 2024) and is moderated by other factors (Hamilton et al., 2015; Shehata et al., 2022), Taylor et al. (2014) found that individuals in the United Kingdom who perceived an increase in extreme weather events, particularly floods and heat waves, had a stronger belief in climate change. Similarly, in Norway, respondents who had observed local environmental changes demonstrated greater awareness of climate issues and greater support for climate action (Lujala and Lein, 2020). Budziszewska’s (2024) qualitative research also revealed that older adults conceptualize climate change based on their personal and local experiences, drawing comparisons between current weather patterns and those of their childhood.

Nevertheless, research on the perceptions and lived experiences of older adults regarding extreme weather is limited, especially in an Austrian context. Therefore, the aim of this study is to investigate whether older adults in Austria perceive changes in weather extremes throughout their lives and which factors correlate with their perception of change.

Several factors that can influence the perception of weather events have been identified in the literature (Cutler, 2015; Hughes et al., 2020; Tenbrink and Willcock, 2023). These are discussed below in order to derive hypotheses.

Individuals differ in how they perceive and recall extreme weather events, and these perceptions are shaped not only by direct experience but also by pre-existing levels of environmental awareness (Myers et al., 2013). Research indicates that interpretations of extreme weather are filtered through cognitive and affective frameworks established prior to the events themselves (Lujala and Lein, 2020; Spence et al., 2012). Myers et al. (2013) demonstrate that individuals with a higher level of environmental engagement are more likely to interpret unusual weather as indicative of broader climate change. In contrast, those with a lower level of engagement tend to make such connections only in response to direct or severe events. In this sense, environmental awareness acts as a perceptual lens directing attention towards environmental cues, shaping the interpretation of observed anomalies and enhancing memory of such events. Consequently, higher environmental awareness increases the likelihood that individuals will perceive changes in extreme weather over time, providing a mechanism through which beliefs about climate change can be formed or reinforced.

*H1*: A higher level of general environmental awareness is associated with the perception of an increase in extreme weather events.

Vulnerability also plays a role in shaping perceptions of environmental change (Clayton, 2020) and can be understood through Adger’s (2006) social-ecological framework, which highlights how vulnerability emerges from the interplay of exposure, sensitivity, and adaptive capacity, mediated by social networks, institutions, and governance structures. Two dimensions of vulnerability should be distinguished for the present study: health and social vulnerability. Older adults with chronic illnesses or limited mobility are more physically and emotionally affected by extreme weather, which can increase their awareness of climatic shifts (Haq and Gutman, 2014; Prina et al., 2024). Conversely, individuals in good health tend to perceive such changes as less severe, as their adaptive capacity and resilience are higher (Rhoades et al., 2018). Social vulnerability may also influence how environmental changes are perceived. For example, socially isolated individuals may have fewer social resources or networks through which environmental risks are discussed and interpreted, which can shape their perceptions of climate related changes (Bixler et al., 2021; Hajek and König, 2022).

*H2*: Older adults who are more vulnerable, either health- or socially, perceive a greater increase in weather extremes since their childhood than those who are in good health.

The place of residence can influence how older individuals experience and interpret weather extremes. Urban environments tend to amplify climatic stress through heat island effects, air pollution, and dense infrastructure (Gabriel and Endlicher, 2011). As a result, extreme heat events may be experienced more intensely in cities, making them more salient in everyday life for urban residents, especially older adults who are more susceptible to heat stress (Antal and Bhutani, 2023). In contrast, rural areas may experience other types of climate-related impacts more strongly, such as droughts, storms, or environmental changes affecting agriculture and local ecosystems. However, these events may be interpreted as part of environmental and seasonal variability rather than as evidence of broader climatic change (Tenbrink and Willcock, 2023). At the same time, studies have found that climate concern and perceived climate risks tend to be lower in rural populations (Mewes et al., 2024). Taken together, these differences in the salience and interpretation of environmental changes suggest that perceptions of increasing weather extremes may differ between urban and rural populations.

*H3*: Older adults living in urban areas are more likely to report an increase in extreme weather events than those living in rural areas.

Financial constraint is another important factor that shapes the perception of weather extremes. Low-income households are often more exposed to environmental hazards due to inadequate housing and limited adaptive resources (Cutler, 2015; Johnson et al., 2012). Baniassadi et al. (2023) demonstrate that people with fewer financial means are more likely to live in densely built urban areas with limited green spaces, making them more vulnerable to heatwaves and flooding. Conversely, wealthier individuals are better equipped to mitigate exposure through air conditioning, insurance, or mobility, which may influence their subjective experience of extreme weather (Baniassadi et al., 2023; de Moura Brito Júnior et al., 2023).

*H4*: Older adults with more financial constraint perceive stronger increases in weather extremes than those with no financial hardship.

To our knowledge, these four hypotheses are being tested for the first time in Austria among older people, using the latest data from SHARE.

## Methods

### Sample design

This study uses data from the Survey of Health, Ageing and Retirement in Europe (SHARE), Wave 9, focusing on Austria. The analysis is mainly based on self-completed drop-off questionnaires administered in addition to the personal interviews (*n* = 3,170). Respondents receive the questionnaire at the end of the interview and complete it independently after the interviewer’s visit. The SHARE drop-off questionnaire is country-specific and varies depending on the wave—data on perceptions of extreme weather events are only available for Austria in wave 9 (SHARE-ERIC, 2024). For the descriptive results, the calibrated cross-sectional individual weights from wave 9 and for the multivariate regression, unweighted data were used (Solon et al., 2015).

Among the Austrian sample, 41% of respondents identify as male and 59% were female. The average age of participants was about 70 years at the time of data collection in 2022. The age distribution shows that 19.5% were under 60 years old, 16.3% were between 60 and 64, 14.7% between 65 and 69, 15.4% between 70 and 74, 13.6% between 75 and 79, and 20.6% were aged 80 or older. Women outnumber men across all age groups, with the largest gender gap in the 80+ cohort. Table 1 provides more descriptive information about the sample.

**Table 1:** Overview of variables and descriptive statistics.

Variables/description		Coding	Distribution	Source
Perception change weather extremes since childhood index		0 = substantial decrease 24 = substantial increase	*M (SUM)* = 18.8389 Std. Dev: 3.2318 6 items	Drop Off Austria
Environmental awareness index		0 = not aware 72 = very aware	*M (SUM)* = 47.3676 Std. Dev: 7.848 18 items	Drop Off Austria
Vulnerability	Subjective health in general	1 = excellent 2 = very good 3 = good 4 = fair 5 = poor	1 = 7.7% 2 = 23.7% 3 = 36.8% 4 = 24.8% 5 = 7.0%	Main Questionnaire
	Loneliness	3 = not lonely 9 = very lonely	*M* = 3.63 Std. Dev: 1.112	Main Questionnaire
Area of living		1 = big city 2 = suburbs of big city 3 = large town 4 = small town 5 = rural area or village	1 = 15.7% 2 = 9.3% 3 = 7.5% 4 = 15.8% 5 = 51.7%	Main Questionnaire
Financial situation		1 = with great difficulty 2 = with some difficulty 3 = fairly easily 4 = easily	1 = 2.3% 2 = 7.8% 3 = 34.7% 4 = 55.3%	Main Questionnaire
Control variables	Education	1 = ISCED 1–2 2 = ISCED 3–4 3 = ISCED 5–6	1 = 19.7% 2 = 51.4% 3 = 28.9%	Main Questionnaire
	Gender	1 = male 2 = female	1 = 41% 2 = 59%	Drop Off Austria
	Age	1 = under 60 2 = 60–74 3 = 75+	1 = 19.5% 2 = 46.3% 3 = 34.2%	Drop Off Austria

### Variables and operationalization

The dependent variable in this study is perception of weather extreme changes since childhood and is based on the following question: “When you think back to your childhood until your 15th birthday, how have the following aspects since then changed, in your opinion?” Respondents rated each of the following items on a five-point scale, ranging from substantially increased (1) to substantially decreased (5): (a) quantity of hot days, (b) quantity and length of droughts, (c) quantity and intensity of storms, (d) quantity and strength of rainfall/flooding, (e) average annual temperature, (f) weather extremes overall. A Principal Component Analysis (PCA) was conducted (eigenvalues ≥ 1 and varimax rotation) to assess the dimensionality of the six indicators of perceived changes in weather extremes (see Appendix). Sampling adequacy was satisfactory (KMO = 0.833), Bartlett’s test confirmed factorability, *χ*^2^(15) = 6150.179, *p* < 0.001 and internal consistency was (Cronbach’s *α* = 0.838) acceptable. Therefore, the six items were summed up to create a continuous index of perceived changes in weather extremes, with higher values indicating stronger perceived increases (ranging from 0 to 24).

All independent variables were selected based on theoretical considerations (see hypotheses), with education, gender, and age additionally included as control variables. Studies show that education (de Bruine Bruin et al., 2025), gender (van der Linden, 2015), and older age groups (Zhang et al., 2025) can also correlate with the perception of extreme weather events.

Environmental awareness was measured using 18 items derived from two sets of questions. Question A assessed environmental values and attitudes with 11 statements rated from 1 (completely agree) to 5 (completely disagree), and Question B included eight statements on support for climate policy measures (e.g., fossil fuel taxation, renewable energy subsidies, and increased adaptation funding) rated from 1 (fully support) to 5 (fully oppose). One item (“Animals should have similar rights as humans”) was excluded based on PCA results (see Appendix). A principal component analysis (PCA) on the remaining 18 items indicated satisfactory sampling adequacy (KMO = 0.771), Bartlett’s test *χ*^2^(153) = 8581.371, *p* < 0.001 and internal consistency was acceptable (Cronbach’s *α* = 0.724). The index ranges from 0 to 72, with higher values indicating greater awareness.

Health and social vulnerability were operationalized using subjective health status and feelings of loneliness. In SHARE, subjective health status is measured using a single item and asks for an overall assessment between 1 (excellent) and 5 (poor). The short version (Hughes et al., 2004) of the R-UCLA scale (Russell et al., 1980) is used to measure loneliness. Respondents answer the three items on a three-point scale. The lowest possible score is 3 (“not lonely”) and the highest is 9 (“very lonely”). In SHARE, older people are asked how they would describe the area of living. The possible answers are big city, suburbs of big city, large town, small town, or rural area or village. This classification was used for the analysis without recoding. Big city was set as the reference category. The financial situation or material circumstances of a household can be measured using various indicators. For the present study, the concept of perceived financial strain was used, which is also applied in EU-SILC. The item asks whether it is possible to make ends meet with the given monthly income. Respondents can answer using a four point scale ranging from ‘with great difficulty’ to ‘easily’.

Education, gender, and age groups were included in the model as control variables. The variable generated in the SHARE dataset according to the ISCED-1997 classification was used to measure education and recoded into three categories. Due to the nonlinearity in the multivariate model age was also categorized under 60, 60–74 and 75 and over. These groups were defined to ensure adequate sample sizes and to align with the average retirement age of around 60 in Austria (OECD, 2019) and the concept of advanced old age (Wahl and Ehni, 2020). These ordinally scaled variables were dummy coded for the statistical model. Gender was also dummy coded, with male gender as the reference category.

### Statistical analysis

Data analysis was conducted using IBM SPSS 29. First, descriptive and bivariate analyses were done to assess distributions and correlations between perceptions of changes in weather extremes and variables stated in the hypothesis. To test the hypothesized influences over and above all other variables, a linear regression model was constructed using perception of changes in weather extremes since childhood as the dependent variable and introducing environmental awareness as well as all mentioned control variables as explanatory variables.

Several diagnostic tests were performed to ensure robustness of the multiple linear regression model. Scatterplots were examined to assess linearity between the dependent and independent variables. Where no clear linear relationship was observed, variables were categorized and recoded as dummy variables. Multicollinearity was evaluated using variance inflation factor (VIF), which ranged from 1.000 to 3.394. These values indicate no evidence of problematic multicollinearity (James et al., 2021). Normality assumption for the dependent variable (perception of changes in weather extremes) and the key predictor (environmental awareness index) was tested using the Kolmogorov–Smirnov and Shapiro–Wilk tests. Both tests rejected the null hypothesis of normality (*p* < 0.001). However, given the large sample size (*N* = 3,110), the Central Limit Theorem ensures the reliability of the regression estimates despite deviations from normality. Durbin–Watson statistic (1.809) confirmed the independence of residuals, indicating no serious autocorrelation. An adjusted *R*^2^ of 0.098 indicates that the included predictors explain approximately 10% of the variance in perceived changes in weather extremes. Additionally, a regression model excluding environmental awareness was estimated and reported in Appendix A (Supplementary Table A3).

## Results

**Figure 1:**
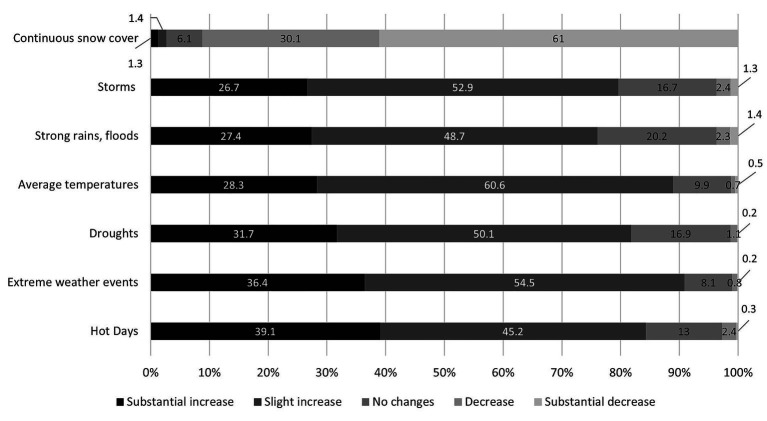
Descriptive results perceptions of changes in weather extremes since childhood.

Figure 1 shows how older adults have perceived changes in weather extremes in Austria since their childhood. The majority reported slight or substantial increases in most weather phenomena. Nearly all respondents observed an increase in hot days (39.1% substantial and 45.2% slight increase), droughts (31.7% substantial and 50.1% slight increase), higher average temperatures (28.3% substantial and 60.6% slight increase) and storms (26.7% substantial and 52.9% slight increase).

The most striking perceived change was the decrease in continuous snow cover, with 91.1% of respondents reporting a decline (61.0% substantial and 30.1% slight). This reflects the awareness of warmer winters in Austria. In contrast, perceptions of heavy rains and flooding were more mixed. Overall, only a very small fraction of respondents perceived decreases in any extreme weather category. In sum, older Austrians overwhelmingly perceive an increase in weather extremes, particularly higher temperatures and reduced snow cover which highlights a shared awareness of climatic shifts over their lifetimes.

Table 2 presents the hierarchical linear regression model used to predict how older adults perceive changes in weather extremes. Higher environmental awareness emerged as the strongest predictor of perceived increases in extreme weather (B = 0.231, *p* < 0.001). The first model explains 5.7% of variance and supports H1. Across all models none of the vulnerability variables (loneliness or subjective health) showed significant effects (all *p* > 0.05) indicating that H2 should be rejected. In model 3 the area of living variables were added and significant differences were found between urban and non-urban groups. Compared to those living in big cities, respondents living in large towns (B = −0.064, *p* = 0.0010), small towns (B = −0.170, *p* < 0.001), and rural areas or villages (B = −0.142, *p* < 0.001) reported perceiving fewer increases in weather extremes, while the coefficient for suburbs of big cities was not significant (B = −0.012, *p* = 0.644). These results support the hypothesis that older people in more rural areas perceive less change in weather extremes compared to those in big cities. Regarding financial situation, respondents who experienced financial difficulty reported significantly higher perceptions of increasing weather extremes (B = 0.054, *p* = 0.017). However, those who reported some difficulty (B = −0.005, *p* = 0.827) or were managing fairly easily (B = −0.011, *p* = 0.619) did not differ significantly from older people who easily make ends meet (all *p* > 0.05). Thus, H4 is partly supported.

**Table 2:** Hierarchical multiple regression model predicting perception of changes in weather extremes.

	Model 1					Model 2					Model 3				
	B (unstd.)	SE	*β* (std.)	t-value	*p*-value	B (unstd.)	SE	*β* (std.)	*t*-value	*p*-value	B (unstd.)	SE	*β* (std.)	*t*-value	*p*-value
Environmental awareness	0.098	0.009	**0.240**	10.89	<0.001	0.098	0.009	**0.241**	10.93	<0.001	0.097	0.009	**0.239**	10.95	<0.001
Health (ref: excellent)															
Very good						0.096	0.274	0.013	0.351	0.725	0.155	0.270	0.022	0.575	0.566
Good						−0.398	0.265	−0.060	−1.50	0.133	−0.302	0.261	−0.046	−1.15	0.247
Fair						0.027	0.281	0.004	0.097	0.923	0.093	0.277	0.012	0.335	0.738
Bad						−0.303	0.384	−0.022	−0.78	0.431	−0.305	0.380	−0.023	−0.80	0.421
Loneliness						0.133	0.070	0.044	1.911	0.056	0.090	0.69	0.030	1.301	0.193
Area of living (ref: big city)															
Suburbs of big city											−0.193	0.279	−0.018	−0.69	0.490
Large town											−0.934	0.305	**−0.076**	−3.05	0.002
Small town											−1.643	0.238	**−0.192**	−6.90	<0.001
Rural area or village											−1.079	0.193	**−0.170**	−5.58	<0.001
Financial situation (ref: easily)															
Fairly easily															
Some difficulty															
Great difficulty															
Education (ref: low)															
Medium															
High															
Gender (ref: m)															
Age (ref: <60)															
60–74															
75+															
Constant	14.395	0.433		33.24	<0.001	14.031	0.54		25.97	<0.001	15.064	0.555		27.15	<0.001
	Adj. *R*^2^ = 0.057, *p* = <0.001					Adj. *R*^2^ = 0.061, *p* = 0.021					Adj. *R*^2^ = 0.088, *p* = <0.001				

	Model 4					Model 5				
	B (unstd.)	SE	*β* (std.)	*t*-value	*p*-value	B (unstd.)	SE	*β* (std.)	*t*-value	*p*-value
Environmental awareness	0.098	0.009	**0.240**	11.02	<0.001	0.094	0.009	**0.231**	10.49	<0.001
Health (ref: excellent)										
Very good	0.156	0.270	0.022	0.577	0.564	0.109	0.270	0.015	0.406	0.685
Good	−0.291	0.261	−0.044	−1.12	0.265	−0.231	0.263	−0.035	−0.88	0.380
Fair	0.076	0.279	0.010	0.273	0.785	0.218	0.283	0.029	0.769	0.442
Bad	−0.385	0.382	−0.028	−1.01	0.314	−0.212	0.386	−0.016	−0.55	0.582
Loneliness	0.092	0.069	0.030	1.329	0.184	0.090	0.070	0.030	1.280	0.201
Area of living (ref: big city)										
Suburbs of big city	−0.159	0.280	−0.015	−0.56	0.569	−0.129	0.279	−0.012	−0.46	0.644
Large town	−0.867	0.306	**−0.071**	−2.83	0.005	−0.786	0.306	**−0.064**	−2.57	0.010
Small town	−1.571	0.240	**−0.183**	−6.55	<0.001	−1.460	0.244	**−0.170**	−5.99	<0.001
Rural area or village	−1.008	0.195	**−0.159**	−5.17	<0.001	−0.901	0.203	**−0.142**	−4.44	<0.001
Financial situation (ref: easily)										
Fairly easily	−0.102	0.150	−0.015	−0.68	0.498	−0.075	0.151	−0.011	−0.49	0.619
Some difficulty	−0.062	0.291	−0.005	−0.21	0.833	−0.064	0.293	−0.005	−0.22	0.827
Great difficulty	1.292	0.500	**0.058**	2.584	0.010	1.199	0.502	**0.054**	2.389	0.017
Education (ref: low)										
Medium						0.296	0.207	0.046	1.429	0.153
High						0.473	0.229	**0.069**	2.061	0.039
Gender (ref: m)						0.278	0.145	0.043	1.916	0.056
Age (ref: <60)										
60–74						0.430	0.185	**0.068**	2.328	0.020
75+						−0.122	0.212	−0.018	−0.58	0.564
Constant	14.985	0.559		26.82	<0.001	14.113	0.645		21.89	<0.001
	Adj. *R*^2^ = 0.091, *p* = 0.051					Adj. *R*^2^ = 0.098, *p* = 0.001				

Education showed a positive association: respondents with a medium (B = 0.046, *p* = 0.153) and a higher level of education (B = 0.069, *p* < 0.039) perceived greater increases in weather extremes than those with a low level of education, although the effect was only statistically significant for people with higher education. Only a trend is evident for gender at a significance level of 0.1: women are more likely than men to report increases in weather extremes (B = 0.043, *p* = 0.056). Concerning age, individuals aged 60–74 reported significantly higher levels of perceived changes in weather extremes than those under 60 (B = 0.068, *p* = 0.039), although the effect size is small. In contrast, no significant difference was observed among respondents aged 75 and over.

## Discussion

The results of this study demonstrate that most older Austrians have perceived an increase in extreme weather since their childhood. This widespread recognition of climate change aligns closely with Ulrich Beck’s thesis of the *risk society*, which posits that modern hazards are global and socially manufactured, creating risks that are ‘democratic’ in perception, even when their consequences are unequally distributed (Beck, 1992). While these perceptions are widely shared, the empirical results of this study indicate that certain factors predict how individuals perceive environmental risks.

The strongest indicator of differences in perceptions of changes in weather extremes is environmental awareness. Respondents with higher environmental awareness were more likely to report increases in weather extremes than no changes or decreases. However, a precise cause-and-effect relationship cannot be determined. Rather, the direction of the relationship remains unclear: it is uncertain whether observed changes in weather extremes make people more certain of the magnitude of negative climate change effects or if existing environmental awareness shapes how people perceive changes in weather extremes. A study in the US showed that both processes can be observed: “Experiential learning” (when personal experiences affect environmental awareness) and “motivated reasoning” (when environmental awareness influences perceptions of changes in weather) (Myers et al., 2013). Experiential learning occurs when individuals with lower engagement levels have personal experiences related to global warming, which leads to increased belief certainty. In contrast, motivated reasoning is more prevalent among highly engaged individuals, whose strong beliefs shape their perceptions of personal experiences. It is likely that these two processes occur: highly aware individuals interpret even minor deviations as evidence of climate change, whereas less engaged older adults update their beliefs primarily following salient or disruptive experiences (Howe and Leiserowitz, 2013). This could serve as a starting point for further research.

Interestingly, our results indicate that health and social vulnerability do not significantly affect the perception of climate change. This is contrary to the expectations based on prior studies that highlighted the susceptibility of older adults to heat waves and storms (Antal and Bhutani, 2023; Haq and Gutman, 2014). However, it is important to distinguish between the perception of changes in the frequency or intensity of extreme weather events, which is captured by the dependent variable in this study, and individuals’ perceptions of their own vulnerability to such hazards. Older adults may recognize that certain events could pose risks to their health without necessarily perceiving that the frequency of these events has changed. In the Austrian context, extreme weather events are not widely perceived as life-threatening dangers from a personal vulnerability standpoint (Wachinger and Renn, 2010). If the perceived threat is absent or unrelated to one’s health or sense of loneliness, then this could explain why awareness is not heightened and why extreme weather is not perceived as more frequent or intense. In contrast, other indicators of vulnerability, such as perceived financial strain, show a significant association with perceptions of change, suggesting that socio-economic factors may shape how environmental changes are interpreted.

A notable pattern also emerged in the urban–rural divide: older city residents were significantly more likely to perceive increases in extreme weather than their rural counterparts, who more frequently reported no change or decreases since childhood. This geographic polarization mirrors findings from Tenbrink and Willcock (2023) and Mewes et al. (2024), who documented lower climate concern in rural populations across Europe. Several factors may explain this trend. Rural environments often mitigate the perceptual impact of extreme events. Forests, soil, and less heat-trapping infrastructure buffer daily climate variations. Additionally, local ecological knowledge may frame extreme weather events as part of long-term cycles rather than as evidence of climate change (Rivero-Romero et al., 2016). Paradoxically, rural seniors scored higher on overall environmental awareness, likely reflecting a strong connection to local ecosystems and a sense of stewardship (Su et al., 2022). The results suggest that awareness and perception are influenced not only by exposure to extreme events but also by cultural, social, and cognitive engagement with the local environment. This divergence between perception and concern illustrates Beck’s (1992) assertion that while risks are universally recognized, the capacity to respond and adapt is socially differentiated.

Testing the socioeconomic variables reveals that older people with higher levels of education perceive more extreme weather events. This suggests that access to information, scientific literacy, and cognitive capacity influence the interpretation of environmental cues (Muttarak and Lutz, 2014). While economic vulnerability is less consistently influential than education, it also plays a role. Respondents who experienced substantial financial difficulty reported higher perceptions of extreme weather. Those with moderate difficulty did not differ significantly from financially secure respondents. This pattern indicates that perceived environmental risks may increase under conditions of material stress. One possible explanation is that economic insecurity increases sensitivity to potential threats and reduces resilience to environmental challenges (Paniello-Castillo et al., 2025).

Looking at the age groups, our results suggest a non-linear relationship between age and perceived changes in weather extremes. Adults aged 60–74 report slightly higher perceptions than those under 60, while the oldest group (75+) does not differ. This aligns with prior research showing that generational differences in climate concern are often stronger for emotional engagement than for cognitive perceptions, with older cohorts sometimes showing lower affective responses but mixed patterns in perceived impacts (Poortinga et al., 2023).

There is also some indication that gender plays a role in shaping climate risk perceptions, in line with arguments that social and structural factors influence climate risk perceptions (van der Linden, 2015). In our study, women tended to be more likely than men to report increases in extreme weather and exhibited higher environmental awareness, although these differences are only weakly significant. This broader pattern is consistent with findings from other industrialized countries, often referred to as the “climate gender gap” (Bush and Clayton, 2023). These disparities can be linked to social roles and systemic inequalities: women often have reduced access to resources, decision-making power, and political influence and are disproportionately affected by climate-related stressors due to responsibilities such as caregiving and household resource management (Apaolaza et al., 2025). Consequently, women’s daily experiences and responsibilities may heighten their sensitivity to environmental changes and their implications for families and communities (Schramkowski and Klus, 2022). Conversely, men may emphasise economic or material concerns, which can reduce their perceived personal risk and shift priorities away from adaptation efforts (Apaolaza et al., 2025). These findings suggest that gender roles and inequalities tend to intersect with environmental risk perception and awareness, shaping how climate threats are noticed, interpreted, and acted upon.

Taken together, the findings demonstrate that awareness and social position expressed through the financial situation, level of education, gender, age and urbanicity influence how climate threats are perceived and addressed (Mewes et al., 2024; Paniello-Castillo et al., 2025; van der Linden, 2015). From a social-ecological perspective (Adger, 2006) these differences reflect how vulnerability emerges from the combination of exposure, sensitivity, and adaptive capacity, which are influenced by social networks and institutions. Additionally, these social and material conditions are filtered through psychological, as explained by construal-level theory, which describes how individuals mentally represent risks that are temporally, spatially, or socially distant (Trope and Liberman, 2010).

## Limitations and conclusion

While this study provides valuable insights into how Austrians aged 50+ perceive weather extremes, it has several limitations. Firstly, the analysis includes a limited set of variables, explaining a small proportion of the variance in perceptions of changes in weather extremes. Some important factors identified in the literature, such as political ideology or environmental behavior, could not be included due to SHARE dataset constraints. Similarly, the multidimensional nature of environmental awareness would have been better captured by incorporating measures of environmental action among older adults, as suggested by other studies (Yang et al., 2024). Additionally, items regarding changes in weather and opinions on climate change were only included in wave 9 of the SHARE drop-off questionnaire in Austria. Consequently, longitudinal dynamics could not be analyzed. It is also important to note that, although a positive association was observed between the perception of changes in weather extremes since childhood and environmental awareness, the direction of causality cannot be determined. Prior research indicates that both processes occur simultaneously (Myers et al., 2013). Furthermore, it must be mentioned that although SHARE aims to be representative of the population aged 50 and older, this study relies on respondents who completed the voluntary drop-off questionnaire. As these respondents may differ systematically from non-respondents (e.g., in health, motivation, or education), the analytical sample may not fully represent the population of older adults in Austria. This potential nonresponse bias should be considered when interpreting the results.

Despite these limitations, the study provides valuable insights into the factors that influence perceptions of environmental change and evidence that older people are aware of these changes. The next step is to transform this awareness into climate-friendly behavior. More research is needed to understand how perceived changes translate into adaptive behavior such as disaster preparedness (Al-rousan et al., 2014). Future research could also adopt a political science perspective and investigate how political ideology shapes perceptions of and engagement with climate policies among the older population. Furthermore, examining the role of media framing in shaping perceptions of extreme weather events could provide valuable insight into how information sources influence environmental awareness among older adults.

However, the results of the present study already demonstrate the necessity of climate adaptation policies to mitigate unequal burdens among specific groups. For older people in Austria it is essential to provide the necessary infrastructure such as climate-resilient housing (Woolrych et al., 2023). Furthermore, fostering intergenerational communities can strengthen social networks and support systems, thereby enhancing collective resilience to climate impacts (Aldrich and Meyer, 2015; Bixler et al., 2021). Empowering older adults through climate education and involvement in climate action initiatives ensures that their needs are met and their voices are heard in urban planning processes.

## Data Availability

The datasets presented in this study can be found in online repositories. The names of the repository/repositories and accession number(s) can be found at: https://doi.org/10.6103/SHARE.w9.900.
