# Supplementary material for: Perceived changes in extreme weather among older people – insights from Austria
Source: Front Psychol. 2026 May 5;17:1794515. doi: 10.3389/fpsyg.2026.1794515 (PMC13183533; doi:10.3389/fpsyg.2026.1794515)
Supplement: Supplementary file 1 [file Data_Sheet_1.docx]

**Appendix A**

**Survey of Health and Ageing in Europe (SHARE): Variables and Index Construction**

**1. Climate Change Perception Index**

The Climate Change Perception Index was constructed from items measuring perceived changes in climate-related phenomena since childhood, including hot days, droughts, storms, floods, average temperatures, extreme weather events, and continuous snow cover.

A principal component analysis (PCA) including all seven items revealed a two-component solution. Six items loaded strongly on the first component (loadings ranging from .705 to .803), whereas the item “continuous snow cover” loaded almost exclusively on a second component (loading = .936) and showed only a negligible loading on the primary component (loading = .063). This indicates that the item captures a distinct dimension not aligned with general perceptions of climate change impacts.

**Table A1: Rotated Component Matrix for Climate Change Perception Items (Seven-Item Solution)**

| **Item** | **Component 1** | **Component 2** |
| --- | --- | --- |
| Climate change – hot days | .71 | .17 |
| Climate change – droughts | .74 | .21 |
| Climate change – continuous snow cover | .06 | .94 |
| Climate change – storms | .74 | -.17 |
| Climate change – strong rains and floods | .74 | -.17 |
| Climate change – average temperatures | .74 | .21 |
| Climate change – extreme weather events | .80 | .12 |

**Note.** Extraction method: principal component analysis. Rotation method: Varimax with Kaiser normalization.

The exclusion of *continuous snow cover* is further supported by its weak correlation with the final index (r = .132, p < .001), indicating limited shared variance.

After excluding this item, PCA with the remaining six items yielded a clear one-component solution. Sampling adequacy was good (KMO = .833), and Bartlett’s test confirmed factorability, χ²(15) = 6150.18, p < .001. The component explained 55.99% of the variance, with loadings between .710 and .801.

The final index was computed by summing the six items minus 6. The index ranges from 0 to 24 Higher values indicate stronger perceptions of climate change impacts. (Cronbach’s α = .838)

**2. Environmental Awareness Index**

Environmental awareness was measured using items from two sets of questions. The first set (Question 11) includes eleven statements on environmental values (1 = completely agree to 5 = completely disagree). The second set (Question 12) includes eight items on support for climate policy measures (1 = fully support to 5 = fully oppose).

Following items were used to construct the environmental awareness index. They are located at the end of the drop off questionnaire after the items about perceptions of extreme weather changes are asked.

Question 11 includes eleven statements that could be answered on a scale ranging from 1= agree completely to 5=disagree completely:

a) Humans have the right to shape nature according to their needs.

b) Plants and animals exist mainly so they can be used by humans.

c) Animals should have similar rights to live as humans.

d) To be able to protect the environment, Austria needs economic growth.

e) There are boundaries of growth which our industrialized world has already surpassed or will soon reach

f) Science and technology will solve many environmental problems without us having to change our lifestyle.

g) We trust science and technology too much and do not trust our feelings enough.

h) Most things that science and technology brought forward hurt the environment.

i) It worries me when I think about under what environmental conditions our children and grandchildren will probably have to live.

j) When I read newspaper articles about environmental problems or watch corresponding TV series, I often feel angry and outraged.

k) If we continue like we have so far, we steer towards an environmental catastrophe.

Out of these eleven items four statements (a, b, d & f) were reversed to be in the same direction as the other ones to measure environmental awareness. Items a and b represent an anthropocentric view that usually opposes environmental consciousness and item f does not recognize the personal responsibility concerning climate change. Statement d can be an ambiguous depending on one’s ideology, but this study takes a degrowth perspective to sustainability.

**What do you think of following measures dealing with climate change?**

Answer scale: 1= agree completely to 5=disagree completely

a) Increases of taxes on fossil fuels (e.g. coal, oil, diesel, paraffin, petrol)

b) Covering part of the energy supply with nuclear energy.

c) Using public funds to promote renewable energy, such as wind power and solar energy.

d) Increasing electricity prices to reduce consumption.

e) Using public funds to promote thermal insulation in residential buildings.

f) Banning the sale of household appliances that are not energy-efficient by law.

g) Spending public funds now to prepare Austria for the impacts of climate change (e.g., expanding flood protection measures).

h) Increasing public funds for developing countries to help them respond to extreme weather events, such as floods and droughts.

All of these items steer into the same direction meaning supporting these measure shows environmental awareness. Again, it has to be recognized that some items can be ambiguous to people answering this question. Statement b for example implies a positive view of nuclear energy, which has been a conflicting topic for Austrians. Through a historical process nuclear energy is associated negatively by a lot of Austrian’s public with environmental and safety issues in mind (Müller et al., 2017).

A PCA including all 19 items revealed a multidimensional structure.

**Table A2**

**Rotated Component Matrix for Environmental Awareness Items (Initial Solution)**

| **Item** | **C1** | **C2** | **C3** | **C4** | **C5** |
| --- | --- | --- | --- | --- | --- |
| Humans have the right to reshape nature according to their needs |  | .73 |  |  |  |
| Plants and animals exist mainly to be utilised by humans |  | .76 |  |  |  |
| Animals should have similar rights as humans |  |  |  |  | .70 |
| To protect the environment, Austria needs economic growth |  | .67 |  |  | -.33 |
| There are limits to growth that our industrial world has surpassed | .60 |  |  |  |  |
| Science and technology will solve environmental problems |  | .58 |  |  |  |
| We trust too much in science and technology | .59 |  |  |  |  |
| Most things created by science and technology are harmful | .62 |  |  |  |  |
| I find it disturbing to think about future environmental conditions | .75 |  |  |  |  |
| I follow environmental problems in the media | .65 |  |  |  |  |
| We are heading for an environmental disaster | .74 |  |  |  |  |
| Increase taxes on fossil fuels |  |  | .83 |  |  |
| Support nuclear energy |  | .44 |  |  |  |
| Public funds for renewable energy |  |  |  | .77 |  |
| Increase electricity fees to reduce energy use |  |  | .77 |  |  |
| Public funds for thermal insulation |  |  |  | .78 |  |
| Ban non-energy-efficient appliances |  |  | .45 |  |  |
| Public funds for climate adaptation |  |  |  | .74 |  |
| Funds for developing countries (climate support) |  |  | .59 | .31 |  |

**Note.** Loadings below .30 are suppressed. Extraction method: principal component analysis. Rotation method: Varimax with Kaiser normalization.

The item “Animals should have similar rights as humans” loaded strongly on a separate component (loading = .696) that was not supported by additional items, resulting in a single-item factor. As factors defined by fewer than three items are considered unstable and difficult to interpret, the item was excluded from further analysis (Abdi & Williams, 2010). Moreover, the item captures a distinct normative dimension (animal rights/biocentrism) that does not align well with the broader construct of environmental awareness and policy support.

Initially, separate PCA components (C1-C4) were extracted, but C3 and C4 were highly correlated, indicating multicollinearity. To account for this and maintain robustness, all items were combined into a single Environmental Awareness Index (Cronbach’s α = .724). The index was constructed by summing all 18 items (after reversing relevant ones) minus 18. The index ranges from 0 to 72

1. **Loneliness**

MH034_companionship (HOW OFTEN LACK COMPANIONSHIP) How much of the time do you feel you lack companionship?

1. Often 2. Some of the time 3. Hardly ever or never

MH035_LeftOut (HOW OFTEN LEFT OUT) How much of the time do you feel left out? Repeat if necessary

1. Often 2. Some of the time 3. Hardly ever or never

MH036_Isolated (HOW OFTEN ISOLATED) How much of the time do you feel isolated from others? Repeat if necessary

1.Often 2. Some of the time 3. Hardly ever or never

The minimum of the resulting score is 3 (“not lonely”) and the maximum is 9 (“very lonely”).

1. **Health**

PH003_HealthGen2 (HEALTH IN GENERAL QUESTION 2) Would you say your health is... Read out.; 1. Excellent 2. Very good 3. Good 4. Fair 5. Poor

1. **Make ends meet (financial situation)**

CO007_AbleMakeEndsMeet (IS HOUSEHOLD ABLE TO MAKE ENDS MEET) Thinking of your household's total monthly income, would you say that your household is able to make ends meet...

1. With great difficulty

2. With some difficulty

3. Fairly easily

4. Easily

1. **Age**

age_int (Age of respondent at the time of interview)

1. **Education**

SHARE is using the International Standard Classification of Education (ISCED), which allows for the standardised reporting of education statistics according to an internationally agreed set of definitions and concepts (for further information see http://uis.unesco.org/en/topic/international-standard-classification education-isced). The gv_isced module contains the 1997 International Standard Classification of Education (ISCED-97).

1. **Gender**

DN042_Gender (MALE OR FEMALE) OBSERVATION Note sex of respondent from observation (ask if unsure) 1. Male 2. Female

1. **Area of Living**

HO037_CityTown (AREA WHERE YOU LIVE) Please look at card 36. How would you describe the area where you live? Read out.; 1. A big city 2. The suburbs or outskirts of a big city 3. A large town 4. A small town 5. A rural area or village

Alternative Model

This following table (A3) presents a regression model excluding environmental awareness.

**Table A3**

**Multiple Linear Regression Model Predicting Perceived Change in Weather Extremes without environmental awareness**

| **Predictor** | **B**  **(Unstd.)** | **SE** | **β**  **(Std.)** | **t-value** | **p-value** |
| --- | --- | --- | --- | --- | --- |
| **Constant** | 18.48 | 0.47 | — | 39.32 | < .001 |
| **Health (ref: excellent)** |  |  |  |  |  |
| very good | 0.05 | 0.26 | .01 | 0.19 | .852 |
| good | -0.29 | 0.25 | -.04 | -1.15 | .252 |
| fair | 0.03 | 0.27 | .00 | 0.11 | .911 |
| poor | -0.20 | 0.35 | -.02 | -0.55 | .579 |
| **Loneliness** | 0.14 | 0.06 | .05 | 2.30 | .021 |
| **Area of living (ref: big city)** |  |  |  |  |  |
| suburbs of big city | -0.19 | 0.26 | -0.02 | -0.71 | .475 |
| large town | -0.87 | 0.28 | -0.07 | -3.07 | .002 |
| small town | -1.59 | 0.23 | -0.18 | -6.89 | < .001 |
| rural area | -1.29 | 0.19 | -0.20 | -6.69 | < .001 |
| **Financial situation (ref: easily)** |  |  |  |  |  |
| Fairly easily | -0.18 | 0.14 | -0.03 | -1.33 | .183 |
| With some difficulty | 0.01 | 0.25 | 0.00 | 0.02 | .985 |
| With great difficulty | 0.47 | 0.46 | 0.02 | 1.02 | .306 |
| **Education (ref: low)** |  |  |  |  |  |
| Middle | 0.42 | 0.18 | 0.07 | 2.39 | .017 |
| High | 0.73 | 0.20 | 0.10 | 3.64 | < .001 |
| **Gender (ref: male)** | 0.39 | 0.13 | 0.06 | 3.02 | .003 |
| **Age group (ref: <60)** |  |  |  |  |  |
| 60–74 years | 0.19 | 0.17 | 0.03 | 1.09 | .276 |
| 75+ years | -0.54 | 0.19 | -0.08 | -2.83 | .005 |
|  | R2= 0.058, VIF range: 1.07–3.76, DW = 1.79 | | | | |

**References**

Abdi, H., & Williams, L. J. (2010). Principal component analysis. *WIREs Computational Statistics*, *2*(4), 433–459. https://doi.org/10.1002/wics.101

Müller, W. C., Müller, W. C., & Thurner, P. W. (Hrsg.). (2017). 98Austria: Rejecting Nuclear Energy—From Party Competition Accident to State Doctrine. In *The Politics of Nuclear Energy in Western Europe* (S. 0). Oxford University Press. https://doi.org/10.1093/oso/9780198747031.003.0005

**Appendix B**

**SPSS Syntax**

** This is a summary of the syntax, showing the preparation of the variables and the statistical procedures that were ultimately performed.

GET

FILE=

'C:\... .

DATASET NAME DataSet1.

**** Clean & Merge ****

DATASET ACTIVATE DataSet1.

FILTER OFF.

USE ALL.

SELECT IF (country=11).

EXECUTE.

DELETE VARIABLES be_fr_q1a to si_q14e_3_eur.

* The following variables must be added:

** sharew9_rel9-0-0_cv_r - variable: age_int

** sharew9_rel9-0-0_gv_isced - variable: isced1997_r

** sharew9_rel9-0-0_gv_housing - variable: areabldgi

** sharew9_rel9-0-0_gv_health - variable: loneliness + sphus

** sharew9_rel9-0-0_co - variable co007 (only hhlevel - must be applied at the individual level)

AGGREGATE

/OUTFILE=* MODE=ADDVARIABLES

/BREAK=hhid9

/co007__first=FIRST(co007_).

** sharew9_rel9-0-0_gv_weights - cciw_w9 (Calibrated cross-sectional individual weight)

***** Preparation of the variables *****

* Perception Change Weather extremes since childhood index

FREQUENCIES at_q10_a to at_q10_g.

MISSING VALUES at_q10_a to at_q10_g (-1, -2).

EXECUTE.

* at_q10_c must be reversed

AUTORECODE VARIABLES=at_q10_c

/INTO at_q10_c_rev

/DESCENDING

/PRINT.

MISSING VALUES at_q10_c_rev (6, 7).

*finale decision: without snow cover item

FACTOR

/VARIABLES at_q10_a at_q10_b at_q10_d at_q10_e at_q10_f at_q10_g

/MISSING LISTWISE

/ANALYSIS at_q10_a at_q10_b at_q10_d at_q10_e at_q10_f at_q10_g

/PRINT INITIAL KMO EXTRACTION ROTATION

/CRITERIA MINEIGEN(1) ITERATE(25)

/EXTRACTION PC

/CRITERIA KAISER ITERATE(25)

/ROTATION VARIMAX

/METHOD=CORRELATION.

RELIABILITY

/VARIABLES=at_q10_a at_q10_b at_q10_d at_q10_e at_q10_f at_q10_g

/SCALE('ALL VARIABLES') ALL

/MODEL=ALPHA.

COMPUTE at_q10_index=(at_q10_a+at_q10_b+at_q10_d+at_q10_e+at_q10_f+at_q10_g)-6.

VALUE LABELS at_q10_index

0 'Substantially increased'

24 'Substantially decreased'.

EXECUTE.

* The index has been reversed for more clarity

RECODE at_q10_index (24=0) (23=1) (22=2) (21=3) (20=4) (19=5) (18=6) (17=7) (16=8) (15=9) (14=10)

(13=11) (12=12) (11=13) (10=14) (9=15) (8=16) (7=17) (6=18) (5=19) (4=20) (3=21) (2=22) (1=23) (0=24)

INTO at_q10_index_rev.

VARIABLE LABELS at_q10_index_rev 'Perception Change Weather extremes since childhood index '.

VALUE LABELS at_q10_index_rev

24 'Substantially increased'

0 'Substantially decreased'.

EXECUTE.

* Environmental awareness index

FREQUENCIES at_q11_a to at_q12_h.

MISSING VALUES at_q11_a to at_q12_h (-1).

EXECUTE.

AUTORECODE VARIABLES=at_q11_c at_q11_e at_q11_g at_q11_h at_q11_i at_q11_j at_q11_k at_q12_a

at_q12_c at_q12_d at_q12_e at_q12_f at_q12_g at_q12_h

/INTO at_q11_c_rev at_q11_e_rev at_q11_g_rev at_q11_h_rev at_q11_i_rev at_q11_j_rev at_q11_k_rev

at_q12_a_rev at_q12_c_rev at_q12_d_rev at_q12_e_rev at_q12_f_rev at_q12_g_rev at_q12_h_rev

/DESCENDING

/PRINT.

MISSING VALUES at_q11_c_rev to at_q12_h_rev (6).

FREQUENCIES at_q11_c_rev to at_q12_h_rev.

*finale decision: without animal rights item

FACTOR

/VARIABLES at_q11_a at_q11_b at_q11_d at_q11_e_rev at_q11_f at_q11_g_rev at_q11_h_rev at_q11_i_rev at_q11_j_rev at_q11_k_rev at_q12_a_rev at_q12_b at_q12_c_rev at_q12_d_rev at_q12_e_rev at_q12_f_rev at_q12_g_rev at_q12_h_rev

/MISSING LISTWISE

/ANALYSIS at_q11_a at_q11_b at_q11_d at_q11_e_rev at_q11_f at_q11_g_rev at_q11_h_rev at_q11_i_rev at_q11_j_rev at_q11_k_rev at_q12_a_rev at_q12_b at_q12_c_rev at_q12_d_rev at_q12_e_rev at_q12_f_rev at_q12_g_rev at_q12_h_rev

/PRINT INITIAL KMO EXTRACTION ROTATION

/CRITERIA MINEIGEN(1) ITERATE(25)

/FORMAT BLANK(.3)

/EXTRACTION PC

/CRITERIA KAISER ITERATE(25)

/ROTATION VARIMAX

/METHOD=CORRELATION.

RELIABILITY

/VARIABLES=at_q11_a at_q11_b at_q11_d at_q11_e_rev at_q11_f at_q11_g_rev at_q11_h_rev at_q11_i_rev at_q11_j_rev at_q11_k_rev at_q12_a_rev at_q12_b at_q12_c_rev at_q12_d_rev at_q12_e_rev at_q12_f_rev at_q12_g_rev at_q12_h_rev

/SCALE('ALL VARIABLES') ALL

/MODEL=ALPHA.

COMPUTE at_q11_12_index_no_c=(at_q11_a + at_q11_b + at_q11_d + at_q11_e_rev + at_q11_f + at_q11_g_rev + at_q11_h_rev + at_q11_i_rev + at_q11_j_rev + at_q11_k_rev +

at_q12_a_rev + at_q12_b + at_q12_c_rev + at_q12_d_rev + at_q12_e_rev + at_q12_f_rev + at_q12_g_rev + at_q12_h_rev)-18.

FREQUENCIES at_q11_12_index_no_c.

* Subjective Health

FREQUENCIES sphus.

SPSSINC CREATE DUMMIES VARIABLE=sphus

ROOTNAME1=sphus_dumm

/OPTIONS ORDER=A USEVALUELABELS=YES USEML=NO OMITFIRST=NO.

* Loneliness

FREQUENCIES loneliness.

DESCRIPTIVES VARIABLES=loneliness

/STATISTICS=MEAN STDDEV MIN MAX.

* Area of Living

FREQUENCIES areabldgi.

SPSSINC CREATE DUMMIES VARIABLE=areabldgi

ROOTNAME1=areabldgi_dumm

/OPTIONS ORDER=A USEVALUELABELS=YES USEML=NO OMITFIRST=NO.

* Financial Situation

FREQUENCIES co007__first.

MISSING VALUES co007__first (-1).

RECODE co007__first (MISSING=SYSMIS).

EXECUTE.

SPSSINC CREATE DUMMIES VARIABLE=co007__first

ROOTNAME1=co007_first_dumm

/OPTIONS ORDER=A USEVALUELABELS=YES USEML=NO OMITFIRST=NO.

* Education

RECODE isced1997_r (1=1) (2=1) (3=2) (4=2) (5=3) (6=3) INTO edu.

VARIABLE LABELS edu "Educational Level (Low=1, Middle=2, High=3)".

VALUE LABELS edu

1 "Low"

2 "Middle"

3 "High".

EXECUTE.

SPSSINC CREATE DUMMIES VARIABLE=edu

ROOTNAME1=edu_dumm

/OPTIONS ORDER=A USEVALUELABELS=YES USEML=NO OMITFIRST=NO.

* Gender

FREQUENCIES gender_do.

* Age

RECODE age_int

(Lowest thru 59 = 1)

(60 thru 74 = 2)

(75 thru Highest = 3)

INTO age_group3.

VARIABLE LABELS age_group3 'age_group3: 1=<60, 2=60-74, 3=75+'.

VALUE LABELS age_group3

1 '<60 years'

2 '60-74 years'

3 '75+ years'.

EXECUTE.

FREQUENCIES age_group3 .

SPSSINC CREATE DUMMIES VARIABLE=age_group3

ROOTNAME1=age_group3_dumm

/OPTIONS ORDER=A USEVALUELABELS=YES USEML=NO OMITFIRST=NO.

**** Analysis ****

** Table 1 **

FREQUENCIES sphus areabldgi co007__first edu gender_do age_group3.

DESCRIPTIVES at_q10_index_rev loneliness at_q11_12_index_no_c.

** Figure 1 ***

WEIGHT by cciw_w9.

FREQUENCIES at_q10_a to at_q10_g.

WEIGHT off.

** Table 2 **

REGRESSION

/MISSING LISTWISE

/STATISTICS COEFF OUTS R ANOVA COLLIN TOL CHANGE

/CRITERIA=PIN(.05) POUT(.10) TOLERANCE(.0001)

/NOORIGIN

/DEPENDENT at_q10_index_rev

/METHOD=ENTER at_q11_12_index_no_c

/METHOD=ENTER sphus_dumm_2 sphus_dumm_3 sphus_dumm_4 sphus_dumm_5 loneliness

/METHOD=ENTER areabldgi_dumm_2 areabldgi_dumm_3 areabldgi_dumm_4 areabldgi_dumm_5

/METHOD=ENTER co007_first_dumm_3 co007_first_dumm_2 co007_first_dumm_1

/METHOD=ENTER edu_dumm_2 edu_dumm_3 gender_do age_group3_dumm_2 age_group3_dumm_3

/RESIDUALS DURBIN.

**Table A3**

REGRESSION

/MISSING LISTWISE

/STATISTICS COEFF OUTS R ANOVA COLLIN TOL

/CRITERIA=PIN(.05) POUT(.10) TOLERANCE(.0001)

/NOORIGIN

/DEPENDENT at_q10_index_rev

/METHOD=ENTER

sphus_dumm_2 sphus_dumm_3 sphus_dumm_4 sphus_dumm_5 loneliness

areabldgi_dumm_2 areabldgi_dumm_3 areabldgi_dumm_4 areabldgi_dumm_5

co007_first_dumm_3 co007_first_dumm_2 co007_first_dumm_1

edu_dumm_2 edu_dumm_3 gender_do age_group3_dumm_2 age_group3_dumm_3

/RESIDUALS DURBIN.
